# Distinct Metabolic Signatures Linked to High-Resolution Computed Tomography Radiographic Phenotypes in Stable and Progressive Fibrotic Lung Disease

**DOI:** 10.3390/metabo16010082

**Published:** 2026-01-19

**Authors:** Girish B. Nair, Faizan Faizee, Zachary Smith, Sayf Al-Katib, Nadia Ashrafi, Ali Yilmaz, Romana Ashrafi Mimi, Sarayu Bhogoju, Vilija Lomeikaite, Juozas Gordevičius, Edward Castillo, Stewart F. Graham

**Affiliations:** 1 Department of Internal Medicine, College of Medicine, University of Kentucky, Lexington, KY 40506, USA; 2 Department of Internal Medicine, Corewell Health William Beaumont University Hospital, Royal Oak, MI 48073, USA; 3 Department of Diagnostic Radiology, Corewell Health William Beaumont University Hospital, Royal Oak, MI 48073, USA; 4 Metabolomics Research, Corewell Health Research Institute, Corewell Health William Beaumont University Hospital, Royal Oak, MI 48073, USA; 5 VUGENE, Grand Rapids, MI 49546, USA; 6 Department of Biomedical Engineering, The University of Texas at Austin, 107 W Dean Keeton St, Austin, TX 78712, USA

**Keywords:** idiopathic pulmonary fibrosis, metabolomics, high-resolution computed tomography, disease progression, biomarkers

## Abstract

**Background**: This study aimed to identify distinct metabolic signatures associated with disease progression by integrating high-resolution computed tomography (HRCT) visual scoring with comprehensive metabolomic profiling. **Materials and Methods**: This single-center, cross-sectional study enrolled 60 idiopathic pulmonary fibrosis/interstitial lung disease (IPF/ILD) patients with usual interstitial pneumonia pattern. Participants underwent standardized pulmonary function testing, HRCT imaging, and peripheral blood collection for metabolomic analysis using one-dimensional hydrogen nuclear magnetic resonance spectroscopy and ultra-high-performance liquid chromatography coupled to tandem mass spectrometry. Linear regression analysis integrated radiographic scores with metabolomic profiles, adjusted for multiple covariates. **Results**: Stable IPF/ILD exhibited moderate negative correlations between the six most significant metabolites and HRCT scores (r = −0.27 to −0.51), along with a high abundance of specific phospholipids (triacylglycerol, monoacylglycerol, phosphatidylglycerol, phosphatidylethanolamine, diacylglycerol), sphingomyelin, ceramide, and acylcarnitine. In contrast, progressive disease showed weak positive correlations between the six most significant metabolites and HRCT scores (r = 0.19–0.26), and moderate negative correlation between specific triacylglycerol species and HRCT scores (r = −0.37–0.4). Furthermore, metabolomic analysis in individuals with progressive disease revealed both high and low abundances of specific phospholipid species (including high and low triacylglycerol species, as well as low levels of phosphatidylglycerol, phosphatidylethanolamine, phosphatidylcholine, phosphatidylserine, and phosphatidylinositol), along with high levels of certain sphingomyelin, ceramide, taurine, and purine bases, and low levels of xanthine and lactic acid observed. **Conclusions**: Integration of systematic HRCT semi-quantitative scoring with metabolomic profiling successfully differentiated stable from progressive IPF/ILD through distinct molecular-radiographic signatures.

## 1. Introduction

Fibrotic interstitial lung disease (F-ILD) encompasses a heterogeneous group of progressive respiratory disorders characterized by pathological extracellular matrix deposition and disruption of normal lung parenchymal architecture [1,2]. While idiopathic pulmonary fibrosis (IPF) represents the archetypal and most severe phenotype among F-ILDs, the distinction between stable and progressive disease trajectories has emerged as a critical prognostic determinant [1]. Progressive F-ILD is distinguished by recurrent acute exacerbations, markedly reduced survival, and substantially increased healthcare utilization and economic burden [1].

High-resolution computed tomography (HRCT) plays a crucial role in diagnosis, with specific attention to the presence of usual interstitial pneumonia (UIP) patterns [2]. This imaging modality enables detailed characterization of pulmonary parenchymal architecture and associated pathological features. HRCT enables detailed evaluation of pulmonary fibrosis through both qualitative and quantitative methodologies. Conventional visual scoring, based on radiologist interpretation, and automated quantitative assessment are widely employed to assess the extent of fibrosis. The prognostic utility of both scoring systems is well-established, with evidence demonstrating their value as independent predictors of disease outcomes [3,4,5,6,7,8,9]. Furthermore, comprehensive study of plasma metabolites has identified distinct metabolic signatures associated with interstitial lung disease progression [10]. These findings suggest that metabolomic profiling could complement imaging-based metrics to enhance prognostication and refine disease stratification [10].

In this cross-sectional study, we investigated the correlation between plasma metabolomic profiles and HRCT, semi-quantitative visual scoring patterns in both stable and progressive F-ILD/IPF cohorts. We hypothesized that specific metabolic signatures would demonstrate significant associations with the extent of radiographic abnormalities, potentially offering novel insights into disease mechanisms and progression. Through multivariate linear regression analysis, we examined the relationship between key metabolites and composite HRCT visual scores, while adjusting for relevant clinical and demographic variables. This investigation represents a crucial step toward integrating radiographic findings with metabolic pathways for enhanced disease characterization/classification and disease in fibrotic lung disease.

## 2. Methods and Materials

### 2.1. Study Design and Patient Cohort

We conducted a single-center, cross-sectional study at Corewell Health William Beaumont University Hospital, Michigan, USA, from December 2021 to October 2022. Out of the 196 participants with ILD/IPF diagnoses who were screened from the ILD Multidisciplinary Clinic, 60 participants with a usual interstitial pneumonia pattern (1. Lower lobe predominant reticulation, 2. Traction bronchiectasis, 3. Honeycombing), who satisfied the inclusion criteria (Supplemental Figure S1) were included in the study.

Institutional Review Board Approval: The study was conducted in accordance with the Declaration of Helsinki and approved by Institutional Review Board of Corewell Health (IRB 2021-327). It was supported by a Supporting Effective Educator Development (SEED) grant. Informed Consent: Informed consent was obtained from all individuals involved in the study. Data Availability: The research data used for this study is available with the supplements.

All enrolled individuals underwent HRCT, standard pulmonary function test, and peripheral blood collection for metabolomic profiling within three months of enrollment. Baseline demographic and clinical data, self-reported sex, self-reported race, medical co-morbidities, body mass index (BMI), among others, were systematically recorded (Supplemental Tables S1–S3).

Individuals were classified as progressive or stable ILD/IPF based on retrospective assessment of clinical trajectory over the preceding 12 months, in line with 2018 IPF and progressive pulmonary fibrosis (PPF) guidelines. Progression was assessed based on the presence of at least two distinct criteria: First, forced vital capacity (FVC) reduction exceeding 10% or diffusion capacity for carbon monoxide reduction (DLCO) reduction exceeding 15%. Second, radiographic advancement of disease affecting greater than 10% of the lung parenchyma. Third, clinical documentation was required to confirm a worsening of respiratory symptoms over the preceding year [11].

### 2.2. Sample Preparation and Quality Control for Metabolomic Analysis

To ensure accurate metabolomic profiling, all blood samples were processed within two hours of collection. Participants underwent an overnight fast for at least eight hours prior to venipuncture, which was performed using serum separator tubes. After collection, blood samples were centrifuged at 1200× *g* for ten minutes at 4 °C, and the resulting serum was promptly aliquoted in cryovials and stored at −80 °C until analysis.

#### 2.2.1. Proton (1H) Nuclear Magnetic Resonance (NMR)

1H NMR Methodology: 1H NMR provides complementary quantitative information on metabolites not routinely measured using mass spectrometry, such as sugar moieties and small organic acids. Serum was filtered through pre-washed (×7) 3.5 KDa filters (Amicon Micron YM-3; Sigma-Aldrich, St. Louis, MO, USA) via centrifugation at 13,000× *g*, at 4 °C for 30 min. To 285 µL of the filtrate, 35 µL of D2O and 30 µL of 11.77 mM sodium 2,2-dimethyl-2-2silapentane-5-sulfonate (DSS) in 50-mmol NaH2PO4 buffer (pH 7) were added. All samples were housed at 4 °C in a thermostatically controlled Sample Jet autosampler (Bruker-Biospin (Manning Park, Billerica, MA, USA)) and heated to room temperature over 3 min prior to analysis by NMR. All 1D 1H NMR data will be randomly collected at 300 (±0.5) K on a Bruker ASCEND HD 600 MHz spectrometer (Bruker-Biospin, Billerica, MA, USA) coupled with a 5 mm TCI cryoprobe. For each sample, 256 transients will be collected as 64 k data points with a spectral width of 12 kHz (20 ppm), using a pulse sequence and inter-pulse delay of 9.65 s.

The data collection protocol included a 180 s temperature equilibration period, fast 3D shimming using the *z*-axis profile of the 2H NMR solvent signal, pulse width calibration, receiver gain adjustment, and acquisition. The free induction decay signal will be zero-filled to 128 k and exponentially multiplied by a 0.1 Hz line broadening factor. The singlet at 0.00 ppm produced by the methyl groups of the internal standard DSS will be used for spectral referencing and quantification. The zero and first order phase constants will be manually optimized after Fourier transformation, and a polynomial baseline correction of the FID (degree 5) was applied for precise quantitation. All spectra will be profiled using Chenomx NMR Suite v9.0 (Edmonton, AB, Canada).

1H NMR Analysis: For 1H NMR analysis, 300 µL of serum was filtered through 3.5 kDa molecular weight cut-off filters, which had been pre-rinsed and centrifuged to remove proteins and macromolecules. The filtrate was then mixed with 28 µL of deuterium oxide (D2O) and 24 µL of 11.77 mM sodium 2,2-dimethyl-2-2silapentane-5-sulfonate in 50-mmol NaH_2_PO_4_ buffer. Spectral acquisition was performed at 310 K using a Bruker Ascend III HD (Manning Park, Billerica, MA, USA) 600.13 MHz spectrometer equipped with a triple resonance cryoprobe. The 1D ^1^H-NMR spectra were obtained using the noesygppr1d pulse sequence, and metabolite identification and quantification were carried out using Bayesil platform [10,11].

#### 2.2.2. Liquid Chromatography-Mass Spectrometry (LC-MS) Workflow

For LC-MS analysis, high-purity solvents were sourced from Fisher Scientific and Sigma Aldrich. Serum aliquots (10 µL) were prepared according to the manufacturer’s protocol (Biocrates Life Sciences AG, Innsbruck, Austria). The preparation involved nitrogen drying, derivatization with phenylisothiocyanate, and extraction in 5 mM ammonium acetate in methanol. Analytical runs were conducted on a Waters I-class UPLC system coupled to a Sciex 7500 (Stryker Corporation, Framingham, MA, USA) mass spectrometer. The Q500 XL kit enabled both direct flow injection analysis and extended load for lipid profiling. Quality control was maintained using three standard levels, with QC2 serving for normalization. QC samples were analyzed at multiple points throughout each batch to monitor instrument performance and ensure data reliability. Data processing and metabolite quantification were performed using MetaboINDICATOR™ within the WebIDQ™ software, allowing for comprehensive calculation of concentrations, metabolite sums, and ratios [10,12].

### 2.3. HRCT Scoring System Development and Implementation

All HRCT examinations were independently evaluated by the thoracic radiologist (S.A.) with over 10 years of experience in interstitial lung disease imaging, who was blinded to clinical data. To mitigate interobserver variability and strengthen reproducibility, all scans underwent an additional independent blinded review by an ILD multidisciplinary group (ILD clinicians); discrepancies were resolved using a prespecified consensus/adjudication process. The scoring system evaluated four cardinal radiographic features: ground-glass opacity (GGO), reticulation, traction bronchiectasis, and honeycombing. Each lung was systematically divided into three zones, yielding six total zones (R1–R3 and L1–L3): (1) Upper zone (R1/L1): apex to carina, (2) Middle zone (R2/L2): carina to inferior pulmonary vein, and (3) Lower zone (R3/L3): inferior pulmonary vein to base. Within each zone, features were scored on a semi-quantitative scale: (0) Absent, (1) Minimal (<5% involvement), (3) Mild (5–25%), (4) Moderate (26–50%), (5) Severe (51–75%), and (6) Very severe (>75%). This systematic approach generated multiple analytical parameters: (1) Individual feature scores, (2) Zonal scores, (3) Cumulative feature scores across zones, (4) Composite zonal scores, and (5) Composite HRCT scores (See Table S3).

### 2.4. Statistical Analysis

All statistical analyses were performed in R. Demographic characteristics, clinical parameters, HRCT composite scores, and metabolite concentrations were compared between groups using nonparametric methods where appropriate; HRCT features are reported as median (IQR) and between-group comparisons were performed using the Wilcoxon rank-sum test. Metabolite concentrations were log2-transformed and quantile-normalized prior to modeling to stabilize variance and reduce technical variability. Differential metabolite analyses and metabolite–HRCT association analyses (performed within disease groups) were conducted using multivariable linear models implemented in the limma package, with models adjusted for HRCT composite visual scores (as applicable to the specific model), age, sex, tobacco exposure (pack-years), BMI, and use of antifibrotic and immunosuppressive therapies; models were fit using lmFit and inference was performed with empirical Bayes moderation via eBayes (robust variance estimation; maximum iterations: 100). To account for multiple testing across metabolites, we controlled the false discovery rate (FDR) using the Benjamini–Hochberg procedure and report q-values (FDR-adjusted *p*-values), which represent the expected proportion of false positives among findings declared significant and are therefore more appropriate than nominal *p*-values in high-dimensional metabolomics [12]. Volcano plots were used solely to visualize the regression results (effect estimates versus statistical significance), and full results are provided in Supplemental Tables S4–S9.

## 3. Results

### 3.1. Demographic and Clinical Characteristics

The median age of patients in the Progressive IPF/ILD disease group (PD) was 75 years, compared to 74 years in the Stable IPF/ILD disease group (SD). Most participants in both groups were self-reported as White (90.9% in PD vs. 92.6% in SD), with a smaller proportion identifying as Black (9.1% in PD vs. 7.4% in SD) (Supplemental Table S1).

Males comprised 54.5% of the PD group compared to 29.6% of the SD group. The prevalence of chronic obstructive pulmonary disease (COPD) was comparable between the groups (15.2% in PD vs. 18.5% in SD). Coronary artery disease (CAD) was more common in the SD group (44.4%) compared to the PD group (30.3%). Similarly, type 2 diabetes mellitus (T2DM) was slightly more prevalent in the SD group (25.9%) compared to the PD group (21.2%). Antifibrotic therapy was more frequently used in the PD group (63.6%) compared to the SD group (44.4%). Immunosuppressive therapy usage was almost similar between the groups (27.3% in PD vs. 29.6% in SD). (Supplemental Table S1). Further, pulmonary function test in progressive disease exhibited lower mean FVC and DLCO values compared to stable disease (Supplemental Table S2).

### 3.2. Comparison of HRCT Composite Scores Between Progressive and Stable Disease Groups

**Figure 1:**
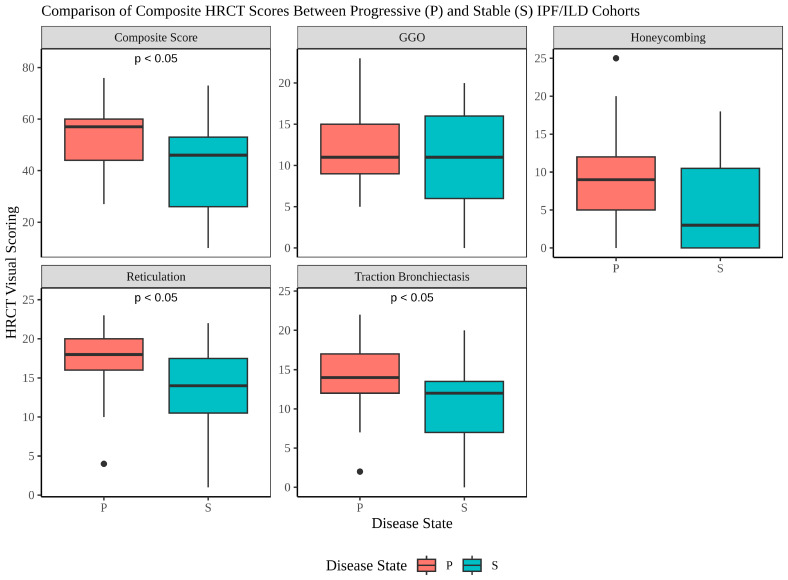
Comparison of HRCT Radiographic Features Between Progressive and Stable Disease Groups. Box plots comparing high-resolution computed tomography (HRCT) visual scoring features between progressive disease groups. Each box represents the interquartile range (IQR) with the horizontal line indicating the median value. Whiskers extend to the most extreme data points within 1.5 times the IQR, and individual points beyond the whiskers represent statistical outliers. Significant differences between P and S groups (*p* < 0.05) are indicated. Ground-glass opacity (GGO), reticulation, traction bronchiectasis, honeycombing, and composite scores were assessed. Reticulation, traction bronchiectasis, and composite scores demonstrated statistically significant differences between the two groups, with higher scores observed in the progressive disease group.

The median and interquartile ranges (IQRs) for each radiological feature, stratified by disease state, are presented in Figure 1. Reticulation scores were significantly higher in the PD cohort compared to the SD cohort (median [IQR]: 18.0 [16.0–20.0] vs. 14.0 [10.5–17.5], *p* = 0.0008). Similarly, Traction Bronchiectasis scores were elevated in the Progressive group (14.0 [12.0–17.0] vs. 12.0 [7.0–13.5], *p* = 0.0129). Composite HRCT scores also demonstrated a significant difference, with higher scores observed in the PD cohort (57.0 [44.0–60.0] vs. 46.0 [26.0–53.0], *p* = 0.0112). No significant differences were observed for GGO or honeycombing scores; however, an upward trend of honeycombing scores was seen in the PD. These findings highlight the distinct radiological features associated with disease progression in IPF/ILD cohorts. Boxplots (Figure 1) illustrate the distribution of radiological features across the two groups, highlighting the significant differences in Reticulation, Traction Bronchiectasis, and Composite Scores.

### 3.3. Metabolomic Correlations with Radiographic Feature in Stable and Progressive IPF/ILD

**Figure 2:**
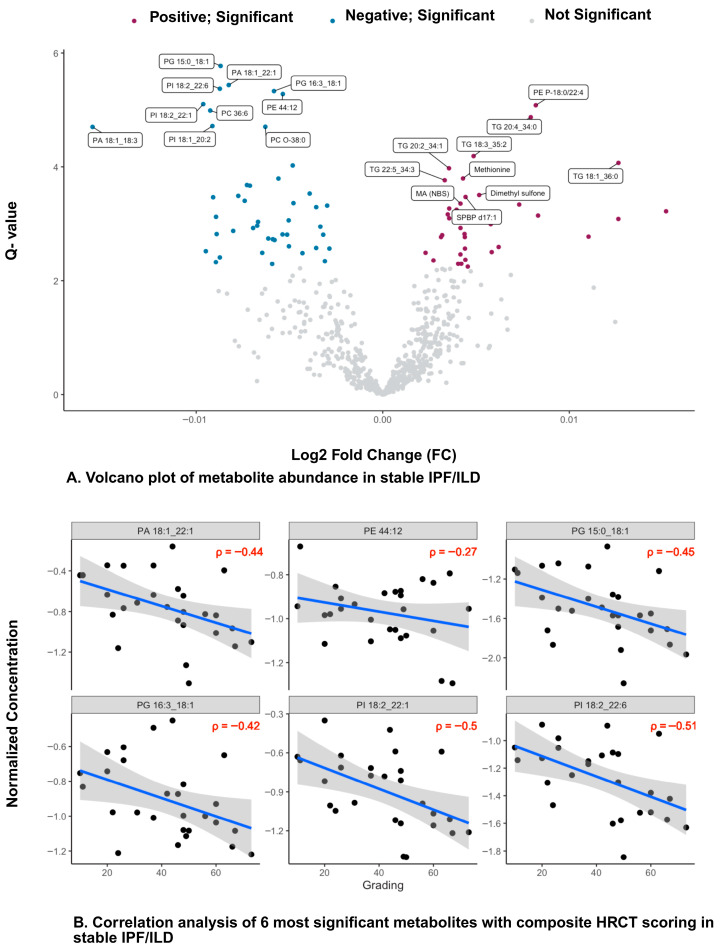

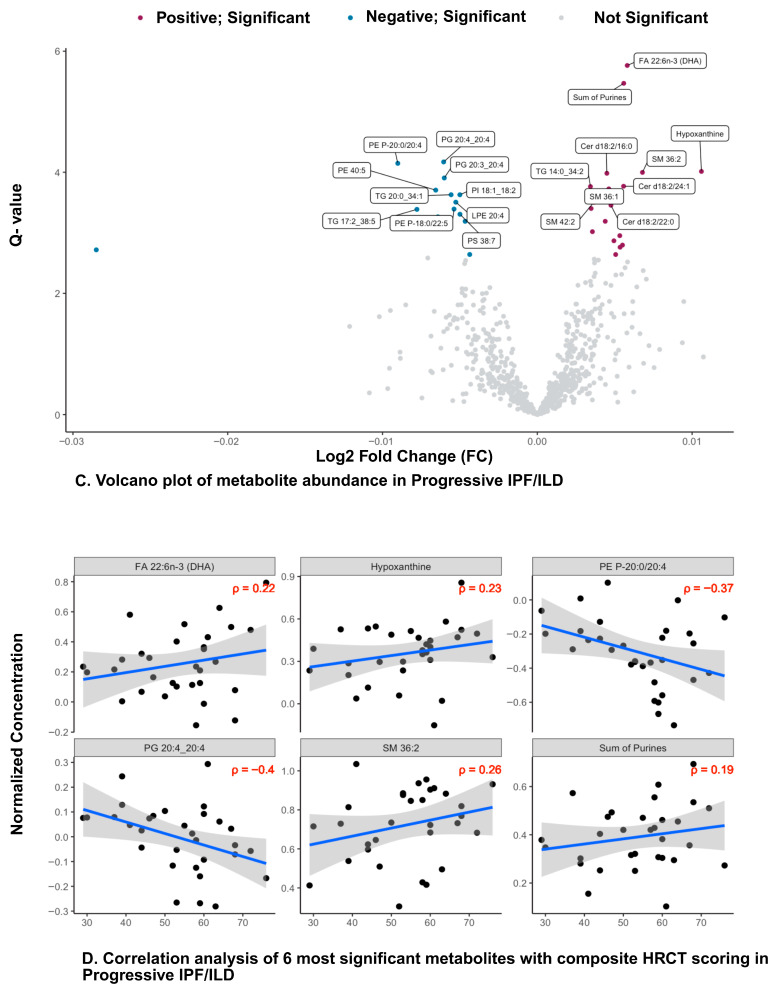
(**A**) Volcano plot of metabolite associations with stable disease. Scatter plot displays metabolites with log fold change (logFC) on the *x*-axis and –log (*p*-value) on the *y*-axis. Metabolites with significant positive relationships (burgundy) and significant negative relationships (teal) are highlighted, with selected metabolites labeled. Non-significant metabolites are shown in gray. (**B**) Correlation analysis of the six most significant metabolites with composite HRCT scores in stable disease. Each panel shows the normalized concentration of a metabolite (*y*-axis) and HRCT grading (*x*-axis). Pearson correlation coefficients (*p*) are indicated. (**C**) Volcano plot of metabolite associations with progressive disease. Scatter plot displays metabolites with log fold change (logFC) on the *x*-axis and −log(*p*-value) on the *y*-axis. Metabolites with significant positive relationships (burgundy) and significant negative relationships (teal) are highlighted, with selected metabolites labeled. Non-significant metabolites are shown in gray. (**D**) Correlation analysis of the six most significant metabolites with composite HRCT scores in progressive disease. Each panel shows the normalized concentration of a metabolite (*y*-axis) versus HRCT grading (*x*-axis). Pearson correlation coefficients (*p*) are indicated.

Linear regression analysis with multiple covariates, including the composite HRCT scoring of four radiographic patterns in stable and progressive disease cohorts, unveiled unique metabolic signatures (Figure 2 and Supplemental Tables S3–S9).

Linear regression analysis with HRCT composite scores in stable IPF/ILD participants revealed significant associations with several metabolites (FDR q-value < 0.05). High-abundant metabolites included several triacylglycerols, diacylglycerol species, monoacylglycerol, phosphatidylethanolamines, phosphatidylinositols, phosphatidylglycerols, sphingomyelin (SM) and ceramides, acylcarnitine, and betaine (FDR q < 0.05; Supplemental Table S8).

In contrast, stable ILD/ILD individuals revealed a significant decrease in the abundance of triacylglycerols, phosphatidylglycerols, phosphatidylethanolamines, phosphatidic acids, lactic acid (Lac), cholesterol ester, and indicators of beta oxidation metabolism (FDR q < 0.05; Supplemental Table S9).

Further, linear regression analysis with HRCT composite scores in progressive IPF/ILD individuals found several differentially significant metabolites (FDR q-value < 0.05). High abundant metabolites included triacylglycerols, a phosphatidylglycerol (PG 16:0_20:3), sphingomyelin and ceramides, hypoxanthine, a fatty acid (FA 22:6n:3 DHA), a cholesterol ester (CE 20.3), taurine, aspartic acid and the sum of purine metabolism (Supplemental Table S5).

Conversely, low abundance metabolites included several triacylglycerols, phosphatidylglycerols, phosphatidylethanolamines, a phosphatidylcholine (LPC 20:4), a phosphatidylserine (PS 38:7), a phosphatidylinositol (PI 18:1_18:2), a bile acid (TMCA), and an indicator of xanthine synthesis metabolism (Supplemental Table S6).

Finally, we performed a Pearson correlation analysis between the top six significantly abundant metabolites and total HRCT scores between progressive and stable ILD/IPF individuals. In stable IPF/ILD individuals, we found moderate negative correlation between significant low abundance metabolites and increasing total HRCT scores: PA 18:1_22:1 (r = −0.4), PE 44:12 (r = −0.27), PG 15:0_18:1 (r = −0.45), PG 16:3_18:1 (r = −0.42), PI 18:2_22:0 (r = −0.5), and PI 18:2_22:6 (r = −0.51) (Figure 2).

In progressive IPF/ILD individuals, we found a weak positive correlation between significant high abundant metabolites and total HRCT scores: FA 22:6n3 DHA (r = 0.22), hypoxanthine (r = 0.23), SM 36:2 (r = 0.26), sum of purine metabolism indicator (0.19). Additionally, a moderate negative correlation was observed between the significantly low-abundant metabolites and total HRCT scores: PE. P 20:0_20:4 (r = −0.37) and PG 20:4_20:4 (r = −0.4).

## 4. Discussion

Our cross-sectional analysis revealed distinct metabolic reprogramming patterns that differentiate stable from progressive fibrotic lung disease through integrated HRCT and targeted metabolomics.

The most clinically significant finding demonstrates meaningful correlations between HRCT composite scores and specific metabolite classes in patients with IPF and other interstitial lung diseases. In progressive disease, we identified weak but statistically significant positive correlations between elevated metabolites and HRCT scores, including fatty acids (r = 0.22), hypoxanthine (r = 0.23), sphingomyelins (r = 0.26), and purine metabolism indicators (r = 0.19). Moreover, moderate negative correlations emerged between decreased phospholipid species and total HRCT scores across both disease states, with correlation coefficients ranging from −0.27 to −0.51. These correlations remained statistically robust after false discovery rate correction and adjusting for several covariates, supporting biologically relevant relationships between systemic metabolic alterations and the extent of structural lung injury quantified by HRCT. The significantly higher composite HRCT scores observed in progressive disease (median 57.0 vs. 46.0, *p* < 0.05) were primarily driven by increased reticulation (18.0 vs. 14.0, *p* < 0.05) and traction bronchiectasis scores (14.0 vs. 12.0, *p* < 0.05). These radiographic differences correspond with distinct metabolic reprogramming patterns, suggesting that the structural features quantified on HRCT reflect underlying metabolic dysfunction that may precede or parallel the morphologic changes visible on imaging.

Both disease groups exhibited alterations across multiple lipid metabolite classes, though with distinct patterns. In stable IPF/ILD, lipid metabolism demonstrates a complex and dynamic profile characterized by extensive TG changes, with several TGs showing increased abundance alongside selective decreases in other TG species, phosphatidylglycerol, and phosphatidic acids. This complex pattern suggests active metabolic compensation mechanisms that may preserve cellular membrane integrity and energy homeostasis. Progressive IPF/ILD exhibited a more constrained lipid signature with limited TG diversity and predominantly decreased abundance across multiple species. This pattern suggests compromised lipid synthetic capacity and suggests a shift toward metabolic pathways that may be insufficient to maintain cellular homeostasis during disease progression.

Progressive disease was characterized by a relatively high abundance of amino acid metabolites, including taurine and aspartic acid, alongside increased purine metabolism indicators and hypoxanthine. Stable IPF/ILD individuals also exhibited evidence of active metabolic processes with increased betaine and acylcarnitine, suggesting ongoing methylation reactions and fatty acid oxidation, though it also shows decreased lactic acid and some acylcarnitine species, suggesting complex alterations in energy metabolism.

The observed dysregulation of lipid and amino acid metabolism aligns with previous reports in IPF/ILD [13,14,15,16]. These metabolic alterations reflect disrupted cellular energetics, as amino acids serve as carbon donors for tricarboxylic acid (TCA) cycle intermediates essential for fatty acid, glucose, and ATP generation [13]. For instance, the conversion of alpha-ketoglutarate to glutamate results in the synthesis of multiple amino acids, including alanine, aspartate, and arginine. Furthermore, branched-chain amino acids (BCAAs; valine, leucine, and isoleucine) can contribute to TCA cycle anaplerosis by generating acetyl-CoA [13,14]. Our findings are concordant with prior studies that have identified dysregulation of amino acids among other metabolites in serum and lung tissue specimens from fibrotic and non-fibrotic IPF/ILD individuals [15,16]. Specifically, a relatively high abundance of serum aspartate in progressors reinforces the potential role of amino acid metabolism in progressive disease [16].

Our metabolomic profiling highlights taurine’s significant metabolic perturbations in the context of progressive fibrotic lung disease. Its observed high abundance is particularly compelling given its established lung-protective mechanisms, including antioxidant and anti-inflammatory actions previously characterized in bleomycin-induced fibrosis models [17,18]. Notably, the positive correlation between purine metabolism, hypoxanthine, fatty acids and HRCT scores suggest that cellular energy metabolism dysfunction parallels the extent of radiographic abnormalities.

Purine metabolites play a crucial role in various cellular processes, including energy metabolism, signaling, and nucleotide synthesis [19]. Moreover, the activation of the mechanistic target of rapamycin complex 1 (mTORC1) signaling pathway, which is sensitive to changes in cellular purine levels [20], has been implicated in the pathogenesis of lung fibrosis [21,22]. Our results revealed low abundance of lactic acid in stable IPF/ILD individuals. Prior studies have shown glycolytic shifts and increased lactate production in fibrotic lung disease, suggesting significant alteration in cellular energy shifts with advanced fibrosis [15,23,24].

Sphingolipid metabolism showed dysregulation, with elevated sphingomyelins and ceramides in progressive disease. From a radiologic perspective, these findings are significant because sphingolipids serve as critical structural membrane components and precursors for pro-apoptotic and inflammatory signaling molecules implicated in fibrotic progression. Further, sphingomyelins are key structural components of cell membranes and precursors for ceramides, which are implicated in apoptosis and pro-inflammatory signaling. These were markedly elevated in both stable and progressive cohorts [25]. The observed disturbance of sphingolipids aligns with prior reports that highlight the role of ceramide and sphingolipid dysregulation in promoting fibrotic and inflammatory processes in pulmonary fibrosis [26,27,28].

Lastly, predominant lipid-centric dysregulation associated with radiographic patterns of fibrosis in our study emerged as a critical hallmark of fibrotic lung disease, characterized by profound alterations in multiple lipid classes, including TGs, CEs, PI, PC, PG, LPCs, ceramides, and SMs. As we mentioned, progressors in our cohort demonstrated more strained lipid dysregulation than the stable disease cohort. There is substantial evidence of aberrant lipid metabolism in fibrotic lung disease, particularly alterations in alveolar epithelial cells (AECs) and the subsequent effects on fibroblast activation and extracellular matrix deposition [29,30,31,32]. All in all, the observed metabolic heterogeneity between stable and progressive IPF/ILD provides critical insights into the molecular underpinnings of disease progression.

This study is the first to correlate HRCT visual scoring with metabolomic profiling, revealing distinct metabolic patterns linked to radiological features, including ground-glass opacities, reticulation, traction bronchiectasis, and honeycombing. By systematically quantifying four cardinal radiographic features across six lung zones, the approach enables detailed, reproducible assessment of disease extent, providing a more precise phenotypic characterization of IPF/ILD. These metabolomic signatures require validation in external cohorts before their potential as predictive or diagnostic tools can be established. Future longitudinal studies will be necessary to determine whether metabolic changes precede radiographic progression. Additionally, we aim to perform targeted lipidomic, sphingomyelin, and amino acid analysis for our future research study to validate our current findings and further delineate pathways of disease progression. The development of targeted therapies aimed at modulating lipids, amino acids, and purine metabolism may hold therapeutic potential for IPF/ILD patients.

Several limitations warrant consideration. The cross-sectional study design with retrospective disease progression assessment limits our ability to establish causal relationships between metabolic alterations and disease progression. Our comparison between stable and progressive groups may reflect differences in disease severity at the time of sampling rather than true progression mechanisms. Another limitation was inclusion of radiographic criterion according to the 2018 progressive fibrotic phenotype and correlating radiographic changes to metabolites. We included both IPF and fibrotic ILD patients with a UIP criteria in the study. Thus, radiographically they had similar patterns and the metabolite differentiation is likely association of progression. Future studies should incorporate longitudinal sampling, tissue-specific metabolomic analyses, and larger cohorts to validate these findings and assess their diagnostic utility.

## 5. Conclusions

Integration of systematic HRCT visual scoring with metabolomic profiling identified distinct associations with a progressive and stable phenotype. The metabolic differences suggest that progressive disease states are associated with heightened amino acid dysregulation (taurine), sphingomyelin, and certain triacylglycerol species. These findings signal toward more active cellular remodeling, inflammation, and energy metabolism shifts in progressive IPF/ILD compared to stable IPF/ILD.

## Data Availability

The original contributions presented in this study are included in the article/Supplementary Materials. Further inquiries can be directed to the corresponding author.

## Supplementary Materials

The following supporting information can be downloaded at: https://www.mdpi.com/article/10.3390/metabo16010082/s1, Figure S1: This flow diagram illustrates the patient selection process for the study. Supplemental Table S1: This table presents the demographic and clinical characteristics of a cohort, divided into two groups; Supplemental Table S2: Pulmonary Function Testing in Progressive and Stable IPF/ILD individuals; Supplemental Table S3: Visual HRCT Scoring in Progressive IPF/ILD and Stable IPF/ILD; Supplemental Table S4: Statistical Analysis of Metabolites Comparing Progressive IPF/ILD with composite HRCT scores; Supplemental Table S5: Relative High Abundant Metabolities (FDR q value < 0.05); Supplemental Table S6: Relative Low Abundant Metabolities (FDR q value < 0.05); Supplemental Table S7: Statistical Analysis of Metabolites Comparing Stable IPF/ILD with composite HRCT scores; Supplemental Table S8: highlights metabolities with significant high abundance (q < 0.05); Supplemental Table S9: highlights metabolities with significant high abundance (q < 0.05).

## Abbreviations

**Lipid Classes**	
**CE**	Cholesterol ester
**Cer**	Ceramide
**DG**	Diacylglycerol
**Hex-Cer**	Hexosylceramide
**LPC**	Lysophosphatidylcholine
**LPE**	Lysophosphatidylethanolamine
**LPI**	Lysophosphatidylinositol
**MG**	Monoacylglycerol
**PA**	Phosphatidic acid
**PC**	Phosphatidylcholine
**PC.O**	Ether-linked phosphatidylcholine
**PE**	Phosphatidylethanolamine
**PE.P**	Plasmalogen phosphatidylethanolamine
**PG**	Phosphatidylglycerol
**PI**	Phosphatidylinositol
**PS**	Phosphatidylserine
**SM**	Sphingomyelin
**SPBP**	Sphingosine-1-phosphate
**TG**	Triacylglyceride
**Amino Acids and Related Compounds**	
**AABA**	α-Aminobutyric acid
**Ala**	Alanine
**Arg**	Arginine
**Asn**	Asparagine
**Asp**	Aspartate
**GABA**	γ-Aminobutyric acid
**Glu**	Glutamate
**Gly**	Glycine
**Phe**	Phenylalanine
**Pro**	Proline
**Ser**	Serine
**Thr**	Threonine
**Trp**	Tryptophan
**Tyr**	Tyrosine
**Val**	Valine
**Metabolic Indicators**	
**CERK**	Ceramide kinase
**CPP**	Carboxypeptidase
**DLD**	Dihydrolipoamide dehydrogenase
**IDO**	Indoleamine 2,3-dioxygenase
**LDH**	Lactate dehydrogenase
**LPIAT1**	Lysophosphatidylinositol acyltransferase 1
**NOS**	Nitric oxide synthase
**OTC**	Ornithine transcarbamylase
**PLA2**	Phospholipase A2
**Fatty Acid Abbreviations**	
**AA**	Arachidonic acid (20:4)
**DHA**	Docosahexaenoic acid (22:6)
**LCFA**	Long-chain fatty acids
**MUFA**	Monounsaturated fatty acids
**PUFA**	Polyunsaturated fatty acids
**UFA**	Unsaturated fatty acids
**VLCFA**	Very long-chain fatty acids
